# Nature-Inspired, Ultra-Lightweight Structures with Gyroid Cores Produced by Additive Manufacturing and Reinforced by Unidirectional Carbon Fiber Ribs

**DOI:** 10.3390/ma12244134

**Published:** 2019-12-10

**Authors:** Marco Pelanconi, Alberto Ortona

**Affiliations:** Mechanical Engineering and Materials Technology Institute (MEMTI), University of Applied Sciences (SUPSI), Via Cantonale 2C, CH-6928 Manno, Switzerland; alberto.ortona@supsi.ch

**Keywords:** sandwich structures, carbon fiber, stereolithography, bio-inspired materials

## Abstract

This article reports on a nature-inspired, ultra-lightweight structure designed to optimize rigidity and density under bending loads. The structure’s main features were conceived by observing the scales of the butterflies’ wings. They are made of a triply periodic minimal surface geometry called gyroid and further reinforced on their outer regions with a series of ribs. In this work, the ribs were substituted with carbon fiber-reinforced bars that were connected to the main structure with an innovative concept. Stereolithography was used to print a plastic component in one piece that comprised the core and the connection system. Bending tests were performed on the structures along with a Finite Element Method optimization campaign to achieve the optimum performance in terms of stiffness and density. Results show that these architectures are among the most effective mechanical solutions in respect to their weight because of their particular arrangement of material in space.

## 1. Introduction

**Figure 1:**
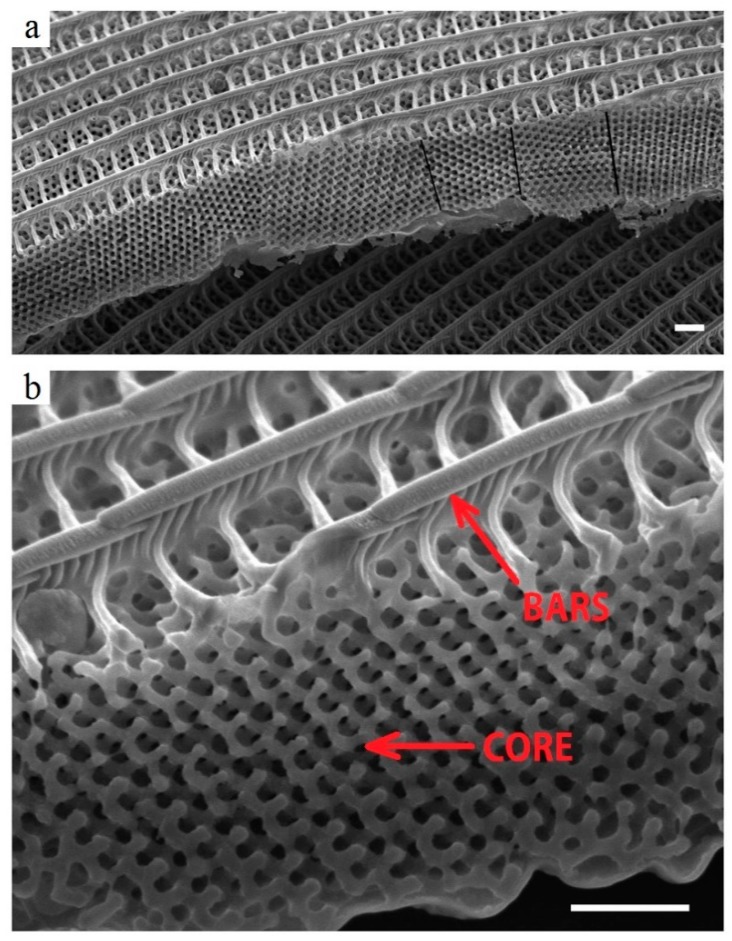
Scanning Electron Microscopy (SEM) micrograph on a cross sectional view of a butterfly wing scale [10]. (**a**) Highly porous central region supported by high-stiff bars. (**b**) The same structure at a higher magnification: Perpendicular smaller fibers make the connection between the porous core and the bars. Scale bar is 1 mm.

Organisms in nature adapt to the surrounding environment thanks to a continuous course of trials-and-errors during which new types of creatures develop and others disappear. This continuous change of characteristics in creatures over time is called evolution. From the mechanical perspective, which is relevant to this paper, evolution has modified parts of living things for their survival. They are structurally optimized according to the contemporary environment, typically to maximize their mechanical properties while minimizing their weight. Nature, however, has the time to perform this optimization work; it is thus obvious that engineers take great inspiration from it [1,2,3,4]. Designers are always striving to better utilize materials in order to find the relative best among several and usually opposed “user’s requirements” [5,6,7]. Several studies inspired by nature have been performed in different application areas, e.g., from the perspective of additive manufacturing, topology optimization, and lightweight structural materials for construction applications [8]. Biological structures such as plant leaves [9] or butterflies wings [10] are natural hybrid materials [11,12] that are made up of multiple components that are combined in specific geometries and scales. Butterfly wings have widely inspired researchers [11,13,14] due to their particular design with several functions such as attracting their mates (optical) or escaping predators (aero-mechanical) [15,16]. Indeed, from a mechanical perspective [10,17,18], their wing can be considered as a structure that is optimized for bending loads. In a cross section of a wing scale, the highly porous central region separates two outer regions that are realized by a frame in which load-bearing bars are connected to the porous core by perpendicular smaller bars (Figure 1). The topology of the inner porous region maximizes the structure’s rigidity while minimizing its weight [10,19,20].

The architecture of the porous structure can be brought back to a gyroid: an infinitely triply periodic minimal surface (TPMS) [20] of relevance in natural science that has been observed in several biological membranes [21]. A gyroid is able to locally minimize its area and free it of self-intersections [22]. It has found several applications in engineering: solar cells, catalytic supports [23], nanoporous membranes, photonic crystals [24], and biomimetic materials [21].

These structures could be designed but hardly manufactured until the advent of additive manufacturing (AM). The great benefit of AM is that it allows the design of components by their function [25,26] and no longer by manufacturability, with an obvious advantage in improving components’ performances [27,28]. AM opens new opportunities for bio-inspired structural components [29,30,31] because living things are built by the most sophisticated of additive manufacturing methods. Among the multiple AM techniques stands stereolithography (SLA) [32], which allows for the fabrication of complex three-dimensional parts with a photopolymerizable resin cured by UV radiation.

In this paper, we present the design, the fabrication, and the characterization of a novel structure with a superior stiffness to weight ratios. We performed our material selection based on the Ashby [6] five inter-related criteria: (i) the function of the structural component that dictates (ii) the choice of materials and their properties, (iii) the shape and size of the components, and (iv) the process used to manufacture the components that interact with (v) the cost and availability (of both material and process). The structure architecture is inspired by butterflies’ wings. By observing their microscale, we further “evolved” the structure by reinforcing the external elements with carbon fiber-reinforced plastic (CFRP) rods. The CFRP rods are connected to the AM structure with epoxy glue. The rest of the structure is composed by a gyroid polymeric core with eternal tubes where the CFRP rods are inserted. This study is meant to validate this new structure’s manufacturing, and it was thus realized for simple bending loads. Further work will be devoted to optimize these new structures for more complex loads.

## 2. Materials and Methods

### 2.1. Design of Ultra-Lightweight Structures

The Computer Aided Design models of the gyroid structures were generated by using Matlab R2017 (MathWorks. Natick, MA, USA). The gyroid cell was designed with plotting a function which reproduces a structure closely similar to the 3D pattern of the gyroid topology:

(1) sinx×cosy+siny×cosz+sinz×cosx=0

**Figure 2:**
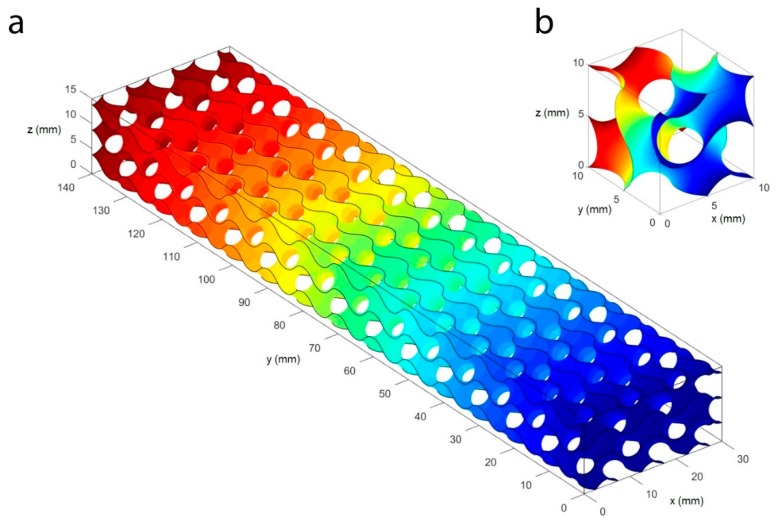
Gyroid surface with cells size of 10 mm. (**a**) 140 × 30 × 15 mm array. (**b**) 10 mm gyroid unit cell.

The unit cell was generated with cell size of 10 mm. The gyroid cell was patterned on the three-dimensional space to the desired core shape with length of 140 mm, a width of 30 mm, and a height of 15 mm. Figure 2 shows the gyroid surface with cells size of 10 mm generated with Matlab R2017. The commercial software Siemens NX 12.0, (Siemens, Munich, Germany) was used to impose the thickness to the surfaces and to export the geometric model to a standard format (STL) adequate for AM purposes. The thickness was one of the parameters analyzed in this study.

**Figure 3:**
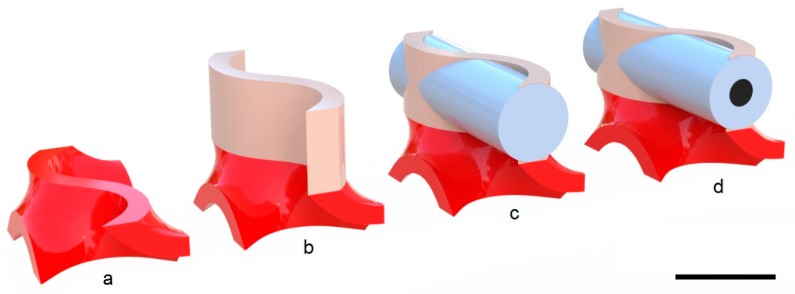
Schematic of the procedure to connect the gyroid with the unidirectional carbon fiber-reinforced plastic (CFRP) inserts. (**a**) Gyroid solid cell with surface thickness of 0.75 mm. (**b**) 3.25 mm extrusion of the cell upper surface. (**c**) Tube addition with diameter of 3.25 mm. (**d**) Addition of the CFRP inserts with a 1.20 mm diameter. Scale bar is 5 mm.

The connection tubes were generated with an external diameter of 3.25 mm and variable internal diameters for different rods. The tubes were connected to the core structure through the connection system shown in Figure 3, which was designed by reproducing the same principle of the butterfly wings scales. The connections were designed by using the commercial CAD software Siemens NX 12.0 (Siemens, Munich, Germany).

**Figure 4:**
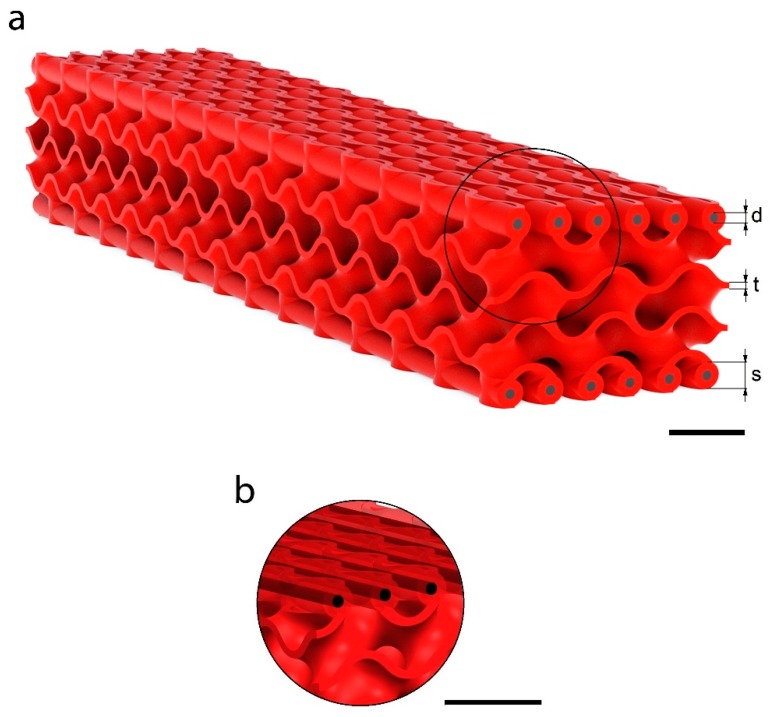
Designed ultra-lightweight structure with gyroid cores and unidirectional CFRP inserts. (**a**) Tri-dimensional CAD model of the designed structure, where “d” is the rods diameter, “t” is the gyroid thickness, and “s” is the rods casing diameter. (**b**) Magnification of the CFRP reinforcements. Scale bar is 10 mm.

Figure 4 shows the final 3D model of the proposed structure with a magnification of the CFRP casings. The models were exported as STL files to perform the AM.

The effects of three variables on the overall performance of the structure were investigated through a parametric study. In Figure 4a, the design parameters are evidenced: gyroid structure thickness (t), CFRP diameter (d), and the casing diameter (s). The size of the CFRP rods corresponded to an approximation of the skin’s thickness in the Ashby graph. This value is crucial in bending applications where sandwich structures are used: The skins are distant from the neutral axis, and they carry most of the load, because they are stiffer than the core. In this condition, the normal stresses are entirely absorbed by the two skins. The core occupies the majority of the volume and its mechanical properties have lower values, but its low density dramatically lowers the weight of the structure. The stress distribution in the panel and its performances are strictly dependent on the ratio between the thicknesses of the skins and the core. This is why the variation of the inserts’ diameter greatly affects the mechanical properties of the entire structure more than the gyroid thickness.

### 2.2. Ashby-Based Approach

**Figure 5:**
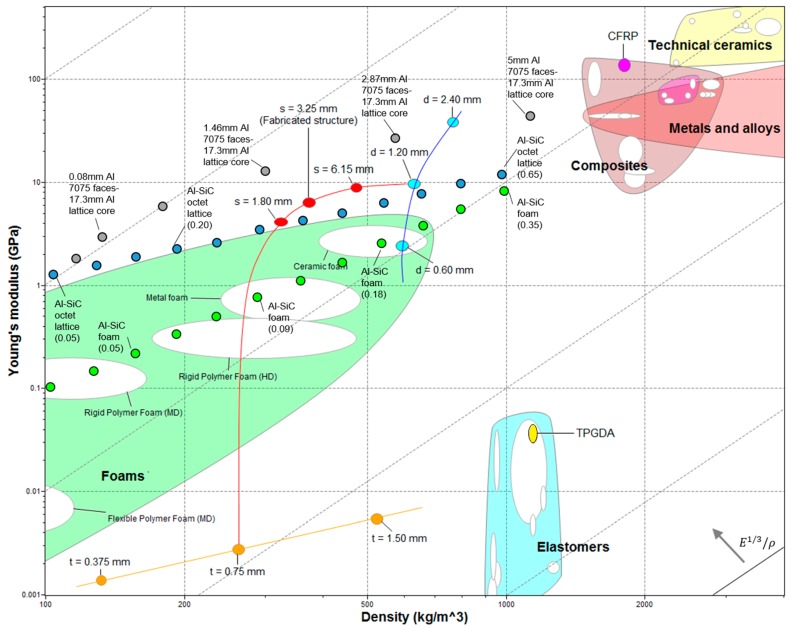
Young’s modulus–density Ashby chart. The chart compares the fabricated structure against other materials. Materials properties were obtained from the Software CES Edupack 2019 (Granta Design, Cambridge, UK).

Figure 5 shows an Ashby chart of the Young’s modulus vs. density. It compares the mechanical properties of the structures in this study, along with its constituents and with other materials. The chart shows the gyroid base elastomeric material, tripropylene glycol diacrylate (TPGDA) (yellow point) and the CFRP (pink point) made up of carbon fibers (63% vol) and epoxy resin (see Section 2.3 for further details regarding the materials used in this study). Table 1 reports the mechanical properties of each constituent.

The three gyroid structure thicknesses (t) selected were: 0.375, 0.75 and 1.50 mm. Knowing the overall size of the core, the cells’ size, and the material’s properties, we calculated porosity, absolute and relative density, and the effective Young’s modulus. The obtained properties were implemented in the Young’s modulus–density Ashby chart (Figure 5, orange dots). The slope values that characterize the lattice materials (so-called slope 1 in Ashby charts) are due to their stretching-dominated behavior [33] (blue dots in the chart). In general, foams show a bending-dominated behavior with a so called “slope 2” in Ashby charts (green dots). The elastic modulus is in accordance with the family of elastomeric materials. The curve shows how the elastic modulus increases by thickening the gyroid structure and therefore increasing the equivalent density of the core. When increasing the gyroid thickness by a factor of 4 (from 0.375 to 1.50 mm), the density and the elastic modulus increase by factors of 3.96 and 3.98, respectively (from 132 kg/m^3^ and 1.40 MPa to 523 kg/m^3^ and 5.58 MPa, respectively).

Three CFRP bars diameters (d) were selected to study the influence of the reinforcement volume fraction on the mechanical properties (0.60, 1.20, and 2.40 mm). The CFRP inserts were joined to the lower and upper side of the gyroid core through a series of innovative connections (Figure 3). The reinforcing bars were inserted into appropriate sheaths co-produced by AM with the core. If we consider, as a first approximation, the distributed CRFP bars plus TPGDA casings as a continuous lamina (as in standard sandwich configurations [6]), then this presents a porosity of about 49% (due to the alternation of voids between the rods; Figure 4b). The materials’ properties obtained were plotted in the Young’s modulus–density Ashby chart (Figure 5, light-blue dots). This new hybrid material configuration is lighter than the CFRP and the TPGDA bulk materials. For the d = 1.20 mm case, the Young’s modulus was reduced with respect to the CFRP by a factor of 14, and it was increased with respect to the TPGDA by a factor of 250. The curve shows how the elastic modulus grew by increasing the insert diameter and therefore the volumetric fraction of the reinforcement material. Increasing the diameter by a factor of 4 (from 0.6 to 2.4 mm), the density and the elastic modulus increases by factor of 1.29 and 15.92, respectively (from 593 kg/m^3^ and 2.45 GPa to 766 kg/m^3^ and 39 GPa, respectively). As expected, the fibers’ volumetric fraction had a higher influence on the rigidity of the structure with respect to the gyroid thickness. This was due to the high elastic modulus of the fibers, for which a small increase in volume led to a large increase in stiffness.

Figure 5 shows the ultra-lightweight structure (red dots) made up of a combination of the gyroid core (orange line) with a 0.75 mm thickness and the reinforced assembly (blue line) with a CFRP diameter of 1.20 mm. The properties of the novel structures were plotted by varying the rods’ casing diameter (s) from the core properties (s = 0 mm, without the reinforcement) to the reinforced assembly properties (s = 7.50 mm, without the core structure). The skins (light-blue dots) are independent in respect to the core thickness (orange dots). All the orange dots can be combined with all the light-blue dots, creating different sandwich structures (red line from an orange to a light-blue dot, for a total of nine combinations).

In this paper, we produced and characterized a porous structure with t = 0.75 mm, d = 1.20 mm and s = 3.25 mm: it showed a density of 373 kg/m^3^ and a Young’s modulus of 6.45 GPa (details are given in the following sections). The structure properties are located (in Figure 5) between those of Al-SiC lattices (blue dots) and Al sandwich (grey dots): Their low density was due to the porous gyroidal core and the relative high elastic modulus with respect to the elastomeric materials is due to their reinforcements. Our structure had higher properties with respect to both foams and lattices made up of Al-SiC [11]. Compared with aluminum sandwich structures, we had a lower modulus due to the material of the core, which was made of an elastomer. This material had a relatively high density and a very low young’s modulus. The proposed structure made up of better performing core materials could significantly improve the mechanical properties.

Figure 4a shows a CAD model of the structure. The three constituents of the structure are shown: (i) a central core, consisting of gyroid unit cell of 10 mm, which is repeated in space, with a volume of 140 × 30 × 15 mm^3^ and (ii) the CFRP bars inside their casings (magnification in Figure 4b). For further CAD modelling details, see the Methods section.

### 2.3. Manufacturing

Among the proposed structures, it was decided to manufacture and test the structure that had a core’s gyroid thickness of 0.75 mm and CFRP inserts’ diameter of 1.20 mm.

The materials employed for the structures were:

(i) A photopolymerizable acrylic resin (Standard Blend Red. FunToDo, Alkmaar, The Netherlands) for the core and the rods casings manufactured in one part;
(ii) CFRP rods Ø = 1.20 mm CFRP rods (Suter Kunststoffe AG, Fraubrunnen, Switzerland), made up of 63vf% mono-directional long carbon fibers and 37vf% epoxy resin for the reinforcement.

The structures were AM manufactured through the stereolithography (SLA) technique. SLA allows for the fabrication of three-dimensional polymeric parts with UV radiation that induces the photopolymerization of a reactive monomer. The structures were manufactured using a 3D printer (GiziMate, Gizmo 3D Printers, Brisbane, Australia) by exploiting the top–down approach [34]. In this process, the STL model is sliced into two-dimensional cross-sections, allowing for their projection in sequence and building the part layer by layer. The platform is immersed in the resin bath up to a distance from the free surface equal to the slice thickness. Upon each projection, the platform is lowered of the same distance. A thickness of 100 µm and an exposure time of 3.5 s were employed for each slice.

**Figure 6:**
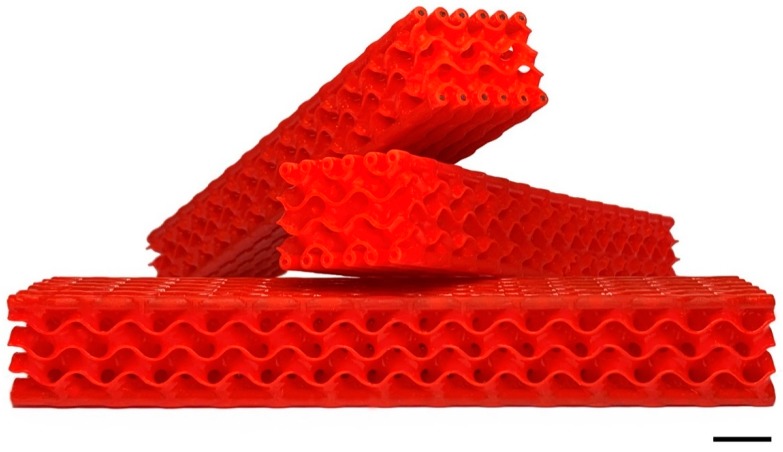
3D printed structure with and without CFRP inserts. Scale bar is 10 mm.

Each polymeric body was subsequently post-cured in a UV oven for 15 min to increase its mechanical strength. Parts were cleaned in an ultrasonic bath with isopropyl alcohol. The CFRP rods were inserted in their casings (0.1 mm larger that the rods) and bonded to them through a thin layer of a two-components epoxy glue (Epoxy-L Härter L. Suter Kunststoffe AG, Fraubrunnen, Switzerland) that was applied to the rods before their insertion. An extra amount of epoxy was expelled during insertion and removed before the cross-linking. The sample produced by AM (Figure 6) weighed 24 g, with a measured density of 373 kg∙m^−3^ and a calculated elastic modulus of 6.5 GPa. For this simple load case, we chose a straight and parallel path of CFRP bars that could be easily modified for more complex loads [9,11,18] following bio-inspired structure designs.

### 2.4. Mechanical Testing

**Figure 7:**
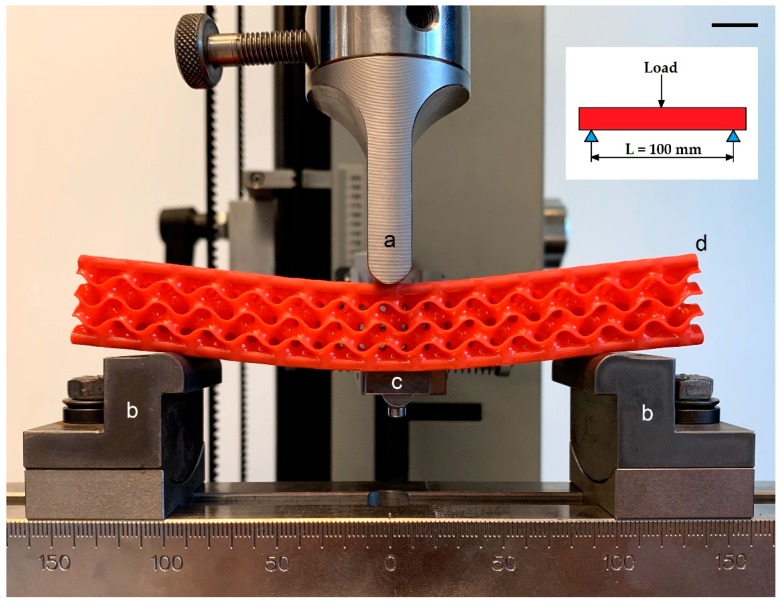
3-point bending tests of the 3D printed structure with CFRP inserts. (**a**) Loading punch. (**b**) Supports with 100 mm distance. (**c**) Extensometer for the central bending deformation. (**d**) Structure in bending. Scale bar is 10 mm. Designation: D7249/D7249M-06.

In order to evaluate the mechanical behavior of the structures, 3-point bending, quasi-static experimental tests were performed with a universal material electromechanical testing machine (Zwick Z050-T1-FR050TH-A1K, Zwick GmbH & Co.KG, Ulm, Germany) at standard conditions (1 atm and 20 °C). Procedures were set according to the designation D7249/D7249M-06. A cell load of 5kN (KAP-S, A.S.T., Dresden, Germany) was used to acquire the reaction force. Structure deformation was measured with a linear variable differential transformer Linear Variable Differential Transformer acquiring the displacements of the lower face of the structure (resolution: ±0.01 mm). The Zwick TestControl tool in the Zwick TestXpert software executed the data acquisition. A span distance of 100 mm and a test speed of 2 mm/min were applied. Two batches of 10 samples each were tested: structures (ii) with CFRP rods and (ii) structures without. Figure 7 shows the experimental 3-point bending apparatus.

### 2.5. Numerical Simulations

A 3D FEM-based approach was followed to simulate the experimental 3-point bending tests on the structures. Therefore, experimental results were compared with the Finite Element Analysis output in the linear-elastic regime until reaching a force of about 35 N (value based on experimental and numerical mechanical characterization of the core material), when simulated with Siemens NX 12.0 Nastran (Siemens, Munich, Germany). Simulations were used to reveal the local stress distribution and to perform a parametric analysis.

The computational domain comprises (i) the 3D structure, (ii) the upper loading support, and (iii) the two bottom supports of the bending equipment. The core was simulated with the gyroid surface (Figure 2) with a thickness of 0.75 mm through the computational grid (mesh). No symmetries were produced due to the non-symmetrical feature of the gyroid cell. This means that the stress distribution in the structure was be symmetrical with respect to the load, even if the test equipment was symmetric. The CFRP rods were simulated as isotropic due to the bending character of the test that allowed for the neglect of the anisotropic character of the material.

As far as the 3D computational domain is concerned, triangular 2D elements were used for the gyroid surface, while hexahedron 3D elements were used for the other bodies. A mesh sensitivity analysis (Table 2) was performed to determine the minimum number of elements that could ensure grid-independent results. Three different computational grids were prepared:

According to the mesh sensitivity results, the normal computational grid was assumed as reference for all the FEM simulations.

The constraints applied to the model were closely related to the experimental set up and test conditions in the linear-elastic regime. The loading punch was set free to move only in Z-direction, and a load of −35 N was implemented to reproduce the experimental negative displacement. The two lower supports were fully constrained. Surface contacts were applied between the supports and the structure (surface-to-surface contact free to move) and between the reinforcements and their arrangements (surface-to-surface gluing). The edge gluing constraint (edge-to-surface gluing) was set between the gyroid structure and the rod casings. The central node of the sandwich structure was constrained in the X- and Y-directions (the isostatic condition of the model) and free to move in the Z direction. The mechanical properties of the structure constituents (see Section 2.3) are reported in Table 1, while the support material (steel in the test) was set as non-deformable.

**Figure 8:**
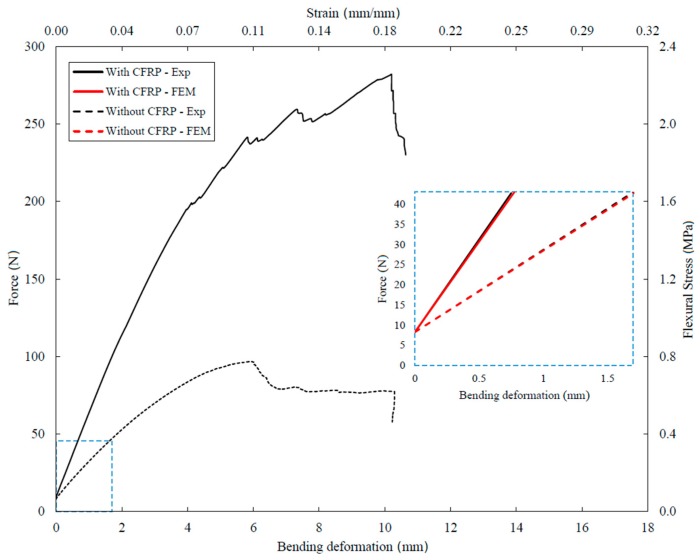
Force–bending deformation and stress–strain chart comparing the experimental and numerical results on the structures with and without CFRP reinforcements. The experimental and numerical curves appear overlapped and the relative errors are below 0.3%.

The validation of the simulation was performed by comparing the bending deformation of the structure with the experimental one under the aforementioned condition (Figure 8). With a 35 N load, the reinforced panel showed an experimental deformation of 0.751 mm and a numerical one of 0.775 mm (relative error: 0.3%).

## 3. Results and Discussion

### 3.1. Bending Tests

Figure 8 shows the force–bending deformation and stress–strain chart that compares the experimental results of the structures with and without CFRP reinforcements. Moreover, it shows the comparison between the experimental and the numerical results in the chosen linear-regime.

The structure with CFRP rods had more than twice the stiffness of the non-reinforced structure (46 ± 4.32 N/mm and 20 ± 3.10 N/mm, respectively) and withstood a maximum load of about 280 ± 10.05 N (100 ± 8.11 N without CFRP). Both structures finally collapsed at 10 mm displacement, pointing out that the collapse was ruled by the core (as happened for sandwich structure with much thinner and rigid skins than the core). However, the reinforced panel reached this condition with a load 3.6 times higher than the non-reinforced one (280 and 77 N, respectively). This was due to the CFRP reinforcements, which were loaded well below their yield strength.

### 3.2. Finite Element Parametric Analysis

The results of the Finite Element model calculation in the linear regime (up to 35 N), showed that: (i) the reinforced panel could support more than twice the load compared to the non-reinforced one; (ii) The CFRP reinforcements underwent a maximum stress of about 186 MPa, while the gyroid structure was stressed with 0.5 MPa (≈0.3% of the maximum value); (iii) The shear stress in the structure of 93 MPa was very high, and it was the half of the von Mises stresses of 186 MPa.

**Figure 9:**
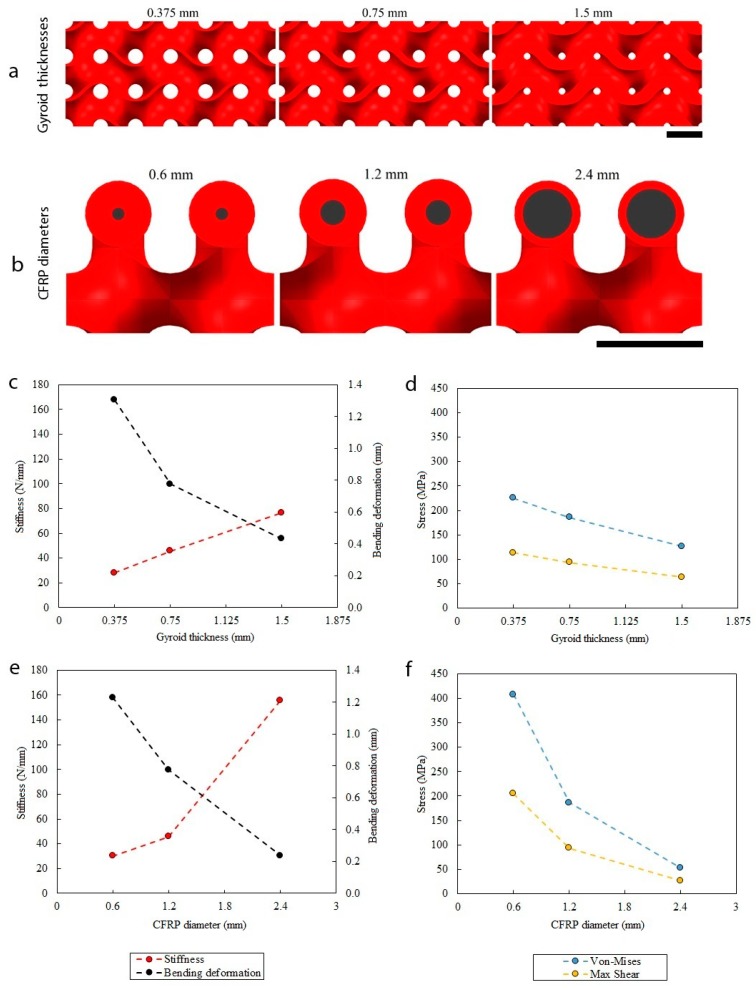
Three-point bending finite element simulations of the structure with different configurations. (**a**) Gyroid thickness variation. (**b**) CFRP diameter variation. (**c**) Stiffness–bending deformation against gyroid thicknesses. (**d**) Stress–gyroid thicknesses chart. (**e**) Stiffness–bending deformation against CFRP diameters. (**f**) Stress–CFRP diameters chart. Scale bar is 5 mm.

**Figure 10:**
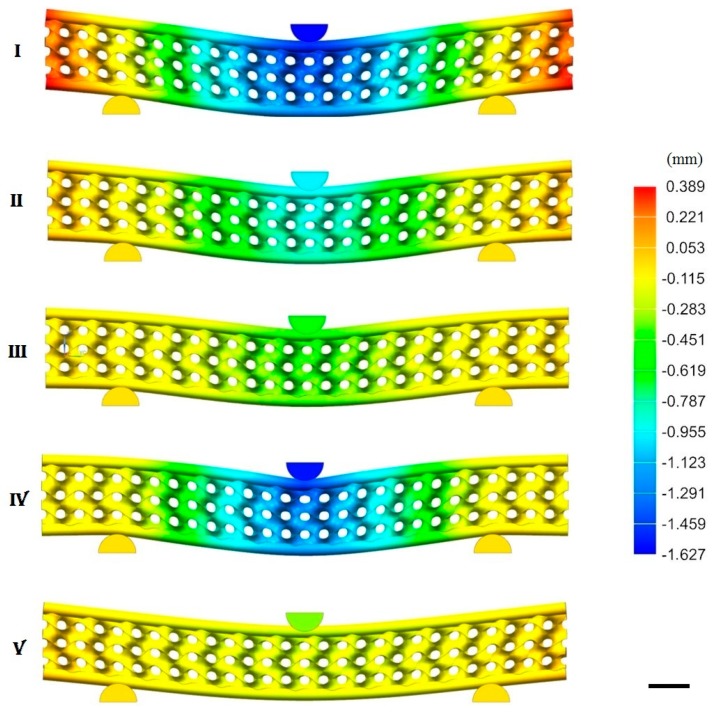
Finite element simulations of the structures: displacement results. (**I**) t = 0.375 mm; d = 1.2 mm. (**II**) t = 0.75 mm; d = 1.2 mm. (**III**) t = 1.5 mm; d = 1.2 mm. (**IV**) t = 0.75 mm; d = 0.6 mm. (**V**) t = 0.75 mm; d = 2.4 mm. t is the gyroid thickness and *d* is the CFRP diameter. Scale bar is 10 mm.

To study the influence on the stiffness and stress distribution in the structure, three gyroid thicknesses (Figure 9a) and three CFRP bars diameters (Figure 9b) were selected. Figure 9c–f shows the parametric analysis results, Figure 10 shows the finite element graphical results of the displacement for different configurations (labelled as cases I, II, ...V), and Table 3 shows the numerical results.

Figure 9c shows the stiffness–bending deformation chart as a function of the gyroid thickness. From the smallest to the largest thickness (case I, II, III), the stiffness increased from 28 to 77 N/mm (+275%), and the deformation decreased from 1.30 to 0.43 mm (−67%). Simultaneously, the von Mises and the shear stresses (Figure 9d) decreased from 225 to 126 MPa (−44%) and from 114 to 63 MPa (−45%), respectively. The increase of stiffness, when a thicker structure was used was related to a decrease of the porosity (from 89% to 54%). Figure 9e shows the stiffness–bending deformation chart as a function of the CFRP diameter. From the smallest to the largest diameter (case IV, II, V), the stiffness increased from 30 to 156 N/mm (+520%), and the deformation decreased from 1.23 to 0.24 mm (−80%). The von Mises and the shear stresses (Figure 9f) decreased from 407 to 53 MPa (−87%) and from 205 to 27 MPa (−87%), respectively. The increase of effective stiffness, when bigger diameters were used, was due to an increase of the high elastic modulus reinforcement volumetric fraction, which increased from 3.4% to 54.5%.

The stiffness constraint of a panel loaded in bending with a central load requires that it must not deflect more than a certain value. The objective is to achieve this with the minimum mass. The well-known material index valid for panels and stiffness performance was used:

(2) $$M=\frac{E^{1/3}}{\rho }$$

The calculated performance index (Table 3) shows that the structure with a gyroid thickness of 0.375 mm and CFRP diameter of 2.4 mm met the optimum (case VII). This configuration had an elastic modulus higher than many other structures (25.8 GPa) and a density of 314 kg∙m^−3^. The resulting performance index was 27.5 GPa^1/3^∙g^−1^∙cm^3^.

## 4. Conclusions

In this article, we propose a new structure that is optimized for bending (commonly called sandwich structure). It comprises an innovative porous core made of gyroid cells and produced by additive manufacturing. The topology of this structure was inspired from the scales of the butterflies’ wings. While the last ones are fully made of organic materials, we enhanced their performance by introducing carbon fiber bars. These bars were connected to the porous core with an innovative solution made possible by the potential of additive manufacturing.

A gyroid surface could be approximated to a trigonometric function and thus easily generated. Thanks to computer-aided design tools, this surface was then transformed into a solid by adding a thickness to it. This approach gave us the possibility to perform a parametric study on the influence of several parameters on the mechanical behavior of the structure (Figure 4).

Some parameters like gyroid thickness do influence the mechanical properties of the bare cores, because adding thickness means adding mass to the structure. This parameter, though, is much less influent when the fiber-reinforced structure is considered. This is because, in general, cores are much less loaded than the skins in a sandwich structure under bending [10]. Indeed, the influence of the CFRP bar diameter is much significant (Figure 9).

One advantage of this solution over the standard sandwich structures is that it directly connects the solid part of the porous core to the mating reinforcing element and thus further minimize its mass. The proposed topological approach can be applied to many materials as long as there is a difference in the elastic modulus between the core and the ribs. Our work aimed at demonstrating the feasibility of this concept, which is why we used the well-known stereo lithography as an AM technique and commercial CFRP rods as ribs. Obviously, metal [35] and ceramic porous periodic architectures [36] can be produced by additive manufacturing, and they can be further reinforced with higher elastic modulus rods.

As an outlook, ad-hoc structures can be designed in order to place the reinforcing rods exactly along the stress lines, as it happens in natural structures like plant leaves [8] and butterfly wings [9].

Nevertheless, from a manufacturing stand point, the present connection concept is limited by the friction of the bar slipping into the plastic tube. This is quite relevant because, in order to achieve a good gluing between the bars and the AM tubes, the gap between the two was kept short. As a consequence, long bars (per se rigid) would be difficult to slide into long tubes with high curvatures.

## Figures and Tables

**Table 1:** Mechanical properties of components.

-	TPGDA	CFRP
ρ (kg∙m^−3^)	1140	1800
E (GPa)	0.0375	140
Poisson’s ratio (-)	0.40	0.28

**Table 2:** Mesh sensitivity results.

-	Coarse	Normal	Refined
Elements (-)	1′642′505	4′895′908	7′242′505
Reaction Z-Force (N)	42.00	35.44	34.95
Maximum VM stress (MPa)	223.90	186.06	184.22
Calculation time (h)	4	7.45	19

**Table 3:** Numerical results of FEA for different configurations.

Case #	I	II	III	IV	V	VI	VII	VIII	IV
Gyroid thickness (mm)	0.375	0.75	1.5	0.75	0.75	0.375	0.375	1.5	1.5
CFRP diameter (mm)	1.2	1.2	1.2	0.6	2.4	0.6	2.4	0.6	2.4
Bending deformation (mm)	−1.31	−0.78	−0.43	−1.23	−0.24	−2.07	−0.40	−0.68	−0.13
Stiffness (N∙mm^−1^)	28.13	45.73	76.69	30.04	155.71	18.48	95.78	50.38	261.13
von Mises stress (MPa)	225.11	186.06	126.42	407.14	53.21	492.59	64.38	276.63	36.15
m (g)	18	24	36	23	27	17	20	35	41
E (GPa)	6.5	6.5	6.5	1.6	25.8	1.6	25.8	1.6	25.8
ρ (kg∙m^−3^)	282	373	555	362	415	274	314	539	617
E^1/3^/ρ (GPa^1/3^∙g^−1^∙cm^3^)	7.7	5.8	3.9	1.5	20.7	2.0	27.5	1.0	13.9
